# Whole-Exome Sequencing Identified Genes Responsible for Thoracic Aortic Aneurysms and Dissections in three Chinese Families

**DOI:** 10.3389/fgene.2022.910932

**Published:** 2022-06-08

**Authors:** Renle Guo, Pengcheng Du, Yifei Pei, Jin Yang, Shuangshuang Li, Sheng Chang, Huiying Sun, Xiaomin He, Jian Dong, Jian Zhou, Zaiping Jing

**Affiliations:** ^1^ Department of Vascular Surgery, Changhai Hospital, Naval Military Medical University, Shanghai, China; ^2^ Department of Vascular Surgery, Taian Central Hospital, Taian, China; ^3^ Department of Vascular Surgery, Suining Central Hostpital, Suining, China; ^4^ Department of Vascular Surgery, Shanghai TCM-Integrated Hospital, Shanghai University of Traditional Chinese Medicine, Shanghai, China

**Keywords:** thoracic aortic aneurysms and dissections, pathogenesis, familial inheritance, whole-exome sequencing, susceptibility gene

## Abstract

Thoracic aortic aneurysms and dissections are precarious conditions that often cannot be diagnosed with fatal outcomes. Over the last few years, pathogenic variants in numerous genes have been identified that predispose to heritable presentations of TAAD. An evidence-based strategy for the selection of genes to test in familial TAAD helps inform family screening and intervention to prevent life-threatening events. Using whole-exome sequencing, four members of three unrelated families clinically diagnosed with TAAD were used to identify the genetic origin of the disorder. Variant evaluation was carried out to detect the pathogenic mutation. Our studies suggest that mutations of *COL3A1* and *ACTA2* are responsible for familial TAAD. In addition, we highlight *FBLN5*, *FBN1*, *SLC2A10*, *FBN2,* and *NOTCH1* as candidate genes. Future studies of crosstalk among the pathways may provide us a step toward understanding the pathogenic mechanism. This finding indicates the necessity of obtaining family medical history and screening of extended relatives of patients with TAAD for the early identification and treatment of TAAD.

## Introduction

Thoracic aortic aneurysms and dissections (TAAD) are one of the major causes of death in humans. TAADs usually grow slowly and quietly to the point of either dissection or rupture, with the most common presenting symptom being death. Non-syndromic TAAD is confined to the aorta and does not have visible clinical signs for early detection (Faggion Vinholo et al., 2019). Non-syndromic TAAD can be classified into familial TAAD and sporadic TAAD according to whether it refers to familial clustering. Familial TAAD is a rare but catastrophic vascular disease; exaggerated changes in components of the aortic wall often cause aorta injury and vascular remodeling. Familial TAADs have a relatively early age of onset and tend to grow at a high rate, exemplifying an aggressive clinical entity. Relatives of TAAD may be seen in the thoracic aorta, abdominal aorta, or cerebral circulation (Albornoz et al., 2006). In affected people, the vascular wall of the aorta may be weakened, stretched, and/or enlarged, which can cause a sudden tear in the aortic intima and lead to blood flowing between the aortic media and adventitia. When blood flow to other parts of the body is reduced and rupture occurs, aortic abnormalities can be life-threatening (Nienaber et al., 2016).

Familial TAAD can be caused by mutations in several genes, and the predominant inheritance pattern is autosomal dominant, with varying degrees of penetrance and expressivity (Keramati et al., 2010). The ClinGen Aortopathy Expert Panel has classified 11 causative genes (*FBN1*, *TGFBR1*, *TGFBR2*, *SMAD3*, *TGFB2*, *COL3A1*, *ACTA2*, *MYH11*, *MYLK*, *LOX*, and *PRKG1*) for HTAAD as category genes in 2018 (Renard et al., 2018). However, even within the same family, the onset of the condition varies from person to person. Conservative or surgical treatment is performed according to the patient’s particular signs and symptoms, and endovascular therapy, open surgery, and hybrid surgery can be used as surgical treatment for different needs. Surveillance of at-risk relatives is often recommended.

Whole-exome sequencing (WES) was an effective and precise method for the identifying causative variants in TAAD families. The analysis of the cost-effective strategy may provide molecular diagnostics, genetic counseling, and individualized health maintenance measures for patients with TAAD (Long et al., 2021). Sufficient knowledge regarding the genetics of hereditary TAADs could feed into the cardiologist’s advice for the best clinical management with appropriate genetic counseling. In the current study, WES was performed on peripheral blood samples of three large 3-generation families, in which multiple members were affected by TAAD. To discover the causative variants in the disease gene, bioinformatics analysis was used to detect the common mutations within the same families. The mutations will be useful for disease risk prediction and management of family members.

## Materials and Methods

### Subject

We recruited three unrelated families with the diagnosis of TAAD from the Department of Vascular Surgery, Changhai Hospital, Naval Military Medical University. All experiments were approved by the ethics committee of Changhai Hospital, Naval Military Medical University. Written informed consent was provided by all participants before participation in this study. Detailed clinical evaluation of each TAAD individual was performed by a cardiologist and a geneticist, including somatoscopy, medical history investigation, and image examination *via* CT angiography (CTA) when necessary. Peripheral blood samples of all TAAD individuals and their family members were collected. According to standard procedures, genomic DNA was extracted for sequencing and molecular analysis. These three families were named TAAD-1, TAAD-2, and TAAD-3. Thirty-seven TAAD-1 patients, 10 TAAD-2 patients, and eight TAAD-3 patients underwent the test of peripheral blood, respectively.

### Whole-Exome Sequencing

Genomic DNA (>1.5 μg for each sample) extracted from peripheral blood was sheared to 200-bp using the Agilent SureSelect Human All Exon v6 (60 Mb) Kit (Agilent Technologies, Santa Clara, CA, United States) and whole exomes were sequenced on the Illumina HiSeq platform (Illumina, San Diego, CA, United States).

### Bioinformatics Analysis

**FIGURE 1:**
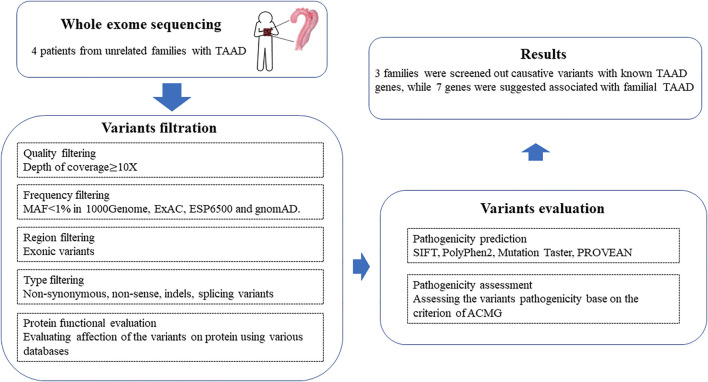
Workflow of bioinformatics analysis in this study.

The workflow of this study is shown in Figure 1. The raw sequencing data were filtered to remove low-quality reads [the ratio of bases with low-quality scores (less than 5) > 50%, ambiguous nucleotide rate >10%, and adapter contamination >5 bp] and then turned into clean reads by Cutadapt (http://code.google.com/p/cutadapt/). Cleaned reads were aligned to the human genome release hg19 using Burrows-Wheeler Aligner (BWA, http://bio-bwa.sourceforge.net/) software. PCR duplicates were marked and removed with Picard (http://picard.sourceforge.net/). After the realignment to the genome, GATK software (https://www.broadinstitute.org/gatk/) was used for base quality recalibration. Somatic SNP/Indel was called by Samtools (bam file process) and GATK (variation calling). Variants obtained from previous steps were annotated with ANNOVAR (Wang et al., 2010) (http://www.openbioinformatics.org/annovar).

First, quality control (QC) including the coverage and average read depth of all coding exons and intron–exon adjacent regions, genotype quality scores, and QualByDepth (Q/D) scores was performed to obtain the variant of high quality. The low-quality variants (read depth <10-fold, quality score <25, Q/D score <5) were excluded from downstream analysis. Next, the remaining variants were filtered according to minor allele frequency (MAF) < 1% in multiple databases, including the 1000 Genome Project (http://browser.1000genomes.org), gnomAD (http://gnomad.broadinstitute.org/), ESP6500, and Exome Aggregation Consortium (ExAC) (http://exac.broadinstitute.org/). In addition, by searching the clinical utility gene card for hereditary TAAD, a TAAD-associated gene list was produced (Arslan-Kirchner et al., 2016) (Supplementary Table S1). The filtered variants were further annotated according to whether they appeared in the TAAD-associated gene list or not. Among the filtered variants, all homozygous and putatively compound heterozygous mutations were picked as candidate TAAD pathogenic variants.

By searching the information in the databases such as ClinVar (Landrum et al., 2020) (http://www.ncbi.nlm.nih.gov/clinvar), cosmic70, and HGMD (Stenson et al., 2003), the candidate variants were annotated with their pathogenicity possibilities. Pathogenic prediction scores were calculated for variants to evaluate the influence of amino acid substitution on protein structure and function with SIFT (Choi and Chan, 2015) (http://sift.jcvi.org), Polyphen2 (Adzhubei et al., 2013) (http://genetics.bwh.harvard.edu/pph2), MutationTaster (Reva et al., 2011) (http://mutationassessor.org), and CADD (Rentzsch et al., 2019) (https://cadd.gs.washington.edu/). The variants were considered pathogenic variants when pathogenic prediction scores were generated by two or more of the tools mentioned above. Finally, mutations that occur in multiple family members are of particular concern. Enrichment analysis on the candidate genes was performed by clusterProfiler to directly investigate in which KEGG pathway or GO term the mutation genes were significantly enriched (Wu et al., 2021; Zhang et al., 2021).

## Results

### Clinical Evaluations

**FIGURE 2:**
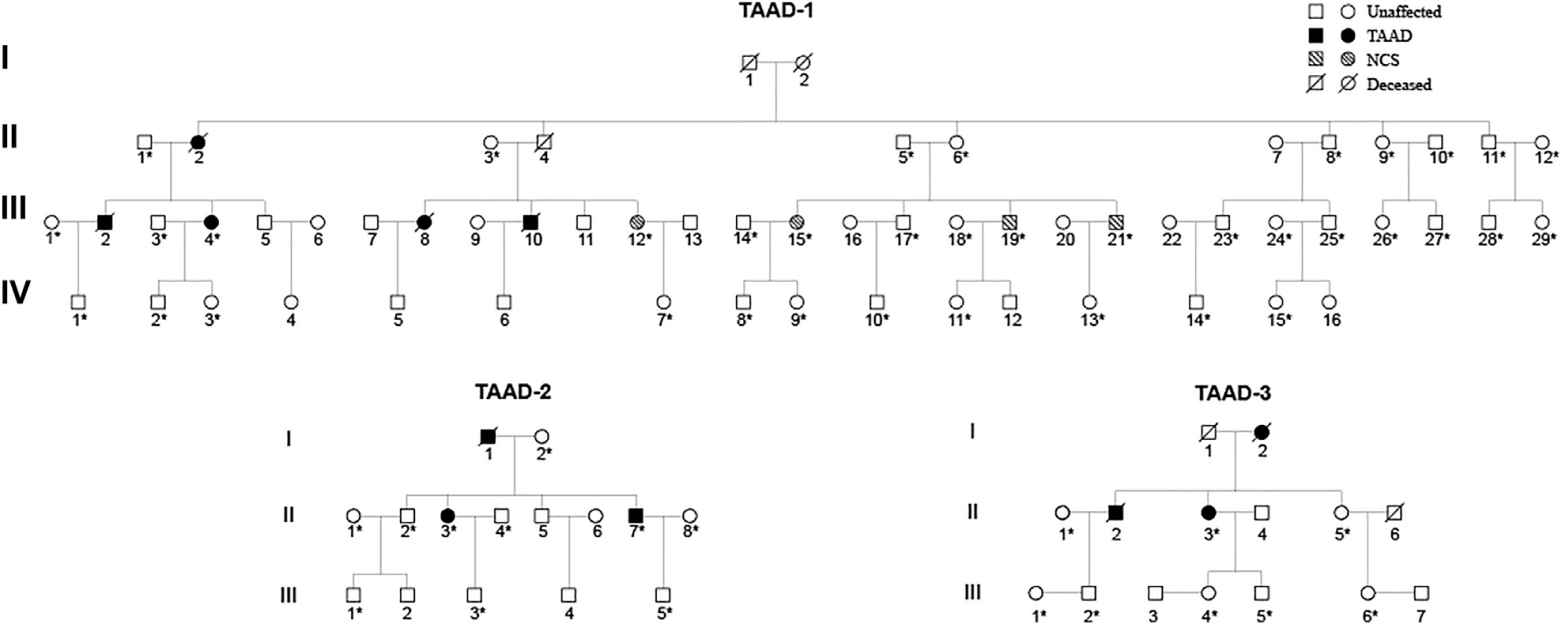
Pedigrees of three unrelated TAAD families: TAAD-1, TAAD-2, and TAAD-3. Square and circle denote male and female, respectively. An asterisk indicates that these samples were whole-exome sequenced.

**FIGURE 3:**
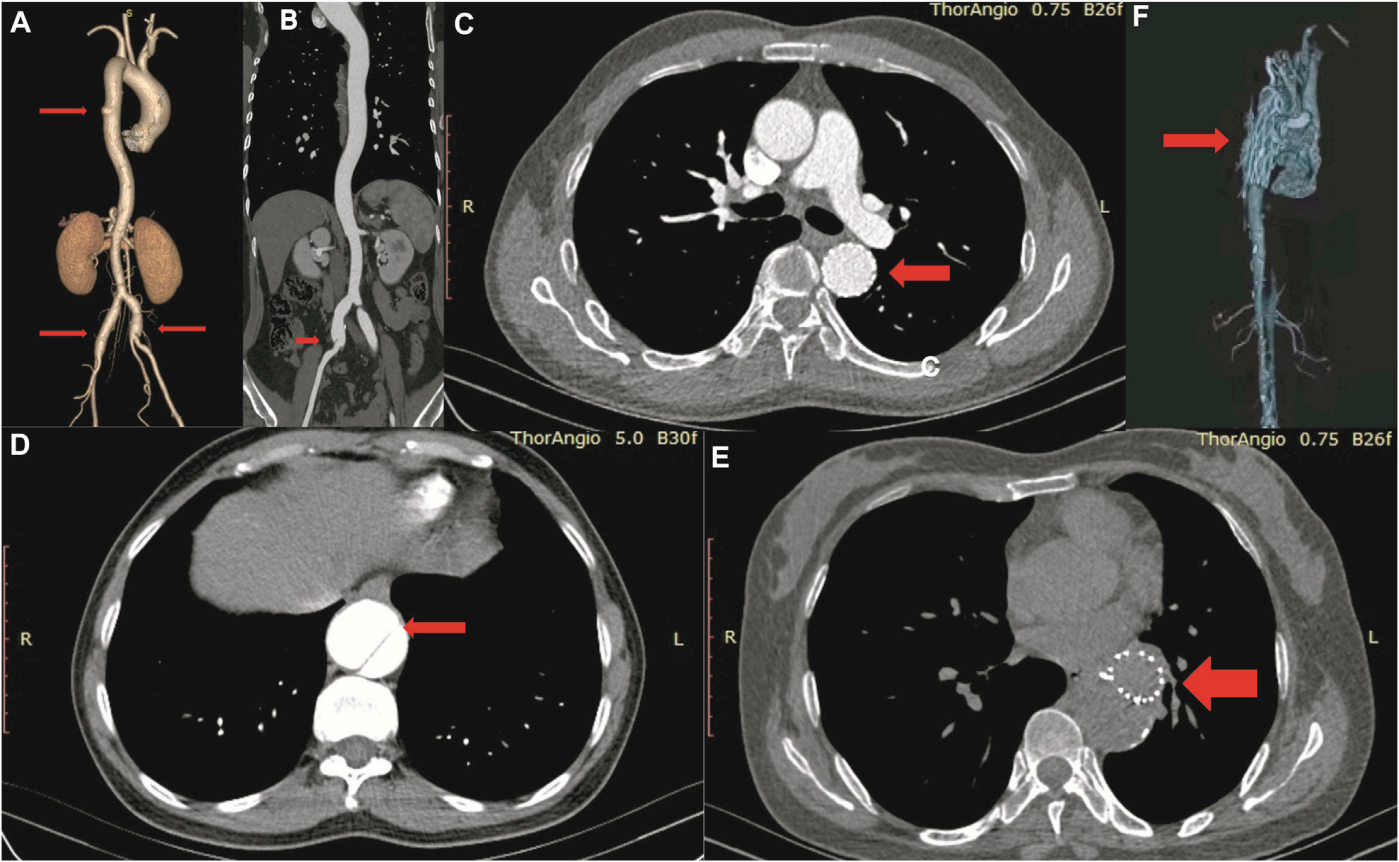
CT angiography image of III-4 in TAAD-1 **(A, B)**, II-3 in TAAD-2 **(C–E)** and II-3 in TAAD-3 **(F)**.

We studied three 3-generation Chinese families (Figure 2). In TAAD-1, the index case (III-4) was a 40-year-old woman who was admitted to the hospital because of abdominal pain for one month. A CTA examination revealed thoracic aortic aneurysm, bilateral common iliac aneurysm (arrow in Figure 3A), and right common iliac artery dissection (arrow in Figure 3B). In addition, III-12, III-15, III-19, and III-21 have been diagnosed with nutcracker syndrome (NCS). NCS is a venous compression syndrome involving the left renal vein. The co-occurrence of TAAD and NCS in the same family indicated the weak and developmental defects of the extracellular matrix (collagen fibers or elastic fibers) in patients, resulting in the decreasing supporting force of the superior mesenteric artery wall, abnormal alignment, and easy collapse.

In TAAD-2, the index case (II-3) was a 45-year-old male patient who was admitted to the hospital due to chest and back pain for 22 h. In the emergency chest and abdomen CTA, thoracic aortic dissection was shown. The condition improved after receiving thoracic endovascular aortic repair. Postoperative check CTA showed the descending aortic stent (arrow in Figure 3C), residual dissection of the proximal abdominal aorta and the dissection tear (arrow in Figure 3D). The index case (II-7) was diagnosed with chronic aortic dissection due to chest and abdominal discomfort for 5 months. After receiving endovascular aneurysm repair, the postoperative check CTA examination revealed the opening true lumen of the celiac trunk and mesentery. Postoperative follow-up CTA showed the descending aortic stent (arrow in Figure 3E). The father of the two patients was suspected to have died of aortic dissection rupture.

In TAAD-3, the index case (II-3) was diagnosed with aortic dissection by CTA due to sudden chest and back pain for 8 h. The Bentall operation was conducted in 2017. The CTA image revealed the reticular stent-like shadow in the aortic arch (arrow in Figure 3F) in the 2021 postoperative review. Below the aortic arch to the distal bifurcation of the abdominal aorta, the formation of true and false lumens can be seen. The patient’s older brother (II-2) was diagnosed with aortic dissection and was discharged from the hospital post endovascular repair of the aortic dissection. Six years after surgery, II-3 died of aortic dissection with rupture despite emergency rescue efforts. The patient’s mother died of suspected aortic dissection.

### Mutation Detection

By filtering the variants for 160 known disease genes (Supplementary Table S1) for TAAD, a total of nine different variants were detected in eight known TAAD genes (Table 1). All these variants are non-synonymous except for the mutation on *SLC2A10*, which is stop gain.

**TABLE 1:** Summary of nine candidate variants in 160 known genes.

Family ID	Sample ID	Gene	Genomic location (hg19) (RefSeq)	Exon	Nucleotide change	Aa change	SIFT	Polyphen2	MAF in 1000 genome
TAAD-1	II-8, III-4^a^, III-12^b^, IV-3, and IV-7	*COL3A1*	chr2:189868799 (NM_000090)	39	c.2753G > A	p.G918E	0	1	—
TAAD-1 (NCS)	Ⅱ-6, II-9,Ⅲ-12^b^, III-15^b^, III-21^b^, and III-26	*COL4A5*	chrX:107865996 (NM_000495)	33	c.2858G > T	p.G953V	0.002	1	0.007947
TAAD-2	I-2, II-3^a^, and II-7^a^	*COL4A5*	chrX:107923924 (NM_000495)	43	c.3940C > T	p.P1314S	0.088	0.606	0.0005298
	II-3^a^, II-7^a^	*FBLN5*	chr14:92336686 (NM_006329)	11	c.1229T > C	p.I410T	0.03	0.763	0.000599
	II-3^a^, II-7^a^, III-3, and III-5	*FBN1*	chr15:48739013 (NM_000138)	47	c.5678A > G	p.N1893S	0.01	0.78	—
	I-2 and II-7^a^	*SLC2A10*	chr20:45353811 (NM_030777)	2	c.136G > T	p.E46X	—	—	—
TAAD-3	II-3^a^, III-2, and III-5	*ACTA2*	chr10:90701142 (NM_001141945/NM_001613)	6	c.460G > A	p.V154M	—	1	—
	II-3^a^	*FBN2*	chr5:127597538 (NM_001999)	64	c.8254G > A	p.D2752N	0.423	0.004	0.0001997
	II-3^a^, III-4, and III-5	*NOTCH1*	chr9:139399931 (NM_017617)	25	c.4417G > A	p.G1473S	0.404	1	—

a Thoracic aortic aneurysms and dissections (TAAD) individual and

b nutcracker syndrome (NCS) individual.

In TAAD-1, *COL3A1* (NM_000090): c.2753G > A, p.G918E was found in five individuals (one TAAD, one NCS, and three normal). The mutation cannot be found in dbSNP. However, mutations in this gene are associated with vascular Ehlers–Danlos syndrome, previously referred to as EDS Type IV. Due to vascular fragility, hemorrhage often occurs in vascular EDS patients that may result in mortality (De Paepe and Malfait, 2012). In addition, the mutation c.2858G > T, p.G953V on *COL4A5* (NM_000495) was found in six individuals (3 NCS, 3 normal). The mutation is rs78972735 in dbSNP and relates to Alport syndrome 1. Alport syndrome (AS) is always accompanied by a spectrum of phenotypes ranging from progressive renal disease with extra renal abnormalities to isolated hematuria. Since the predominant inheritance pattern of TAAD is autosomal-dominant, the mutation of *COL4A5* is not responsible for TAAD-1.

In TAAD-2, three mutations were present in both index cases II-7 and II-3. *COL4A5* (NM_000495): c.3940C > T, p.P1314S were found in three individuals (two TAAD and one normal). The mutation was not recorded in dbSNP. *FBLN5* (NM_006329): c.1229T > C, p.I410T were found in two TAAD individuals. Mutations in *FBLN5* are associated with age-related macular degeneration 3, cutis laxa. *FBN1* (NM_000138): c.5678A > G, p.N1893S were found in four individuals (two TAAD and two normal). Mutations in NM_000138 are associated with geleophysic dysplasia, familial TAAD, and Marfan syndrome. Moreover, *SLC2A10* (NM_030777): c.136G > T, p.E46X was found in two individuals (one TAAD and one normal). Mutations in NM_030777 are associated with cardiovascular phenotype, familial TAAD, and arterial tortuosity syndrome.

In TAAD-3, *ACTA2* (NM_001141945/NM_001613): c.460G > A, p.V154M were found in three individuals (one TAAD and two normal). Mutations in NM_001141945 are associated with aortic aneurysm, familial thoracic six, and multisystemic smooth muscle dysfunction syndrome. In *FBN2* (NM_001999): c.8254G > A, p.D2752N were found in the TAAD individuals. Mutations in NM_001999 are associated with congenital contractual arachnodactyly and cardiovascular phenotype. *NOTCH1* (NM_017617): c.4417G > A, p.G1473S, were found in three individuals (one TAAD and two normal). Mutations in NM_017617 are associated with cardiovascular phenotype, Adams-Oliver syndrome 5, and familial TAAD.

### Enrichment Analysis

**FIGURE 4:**
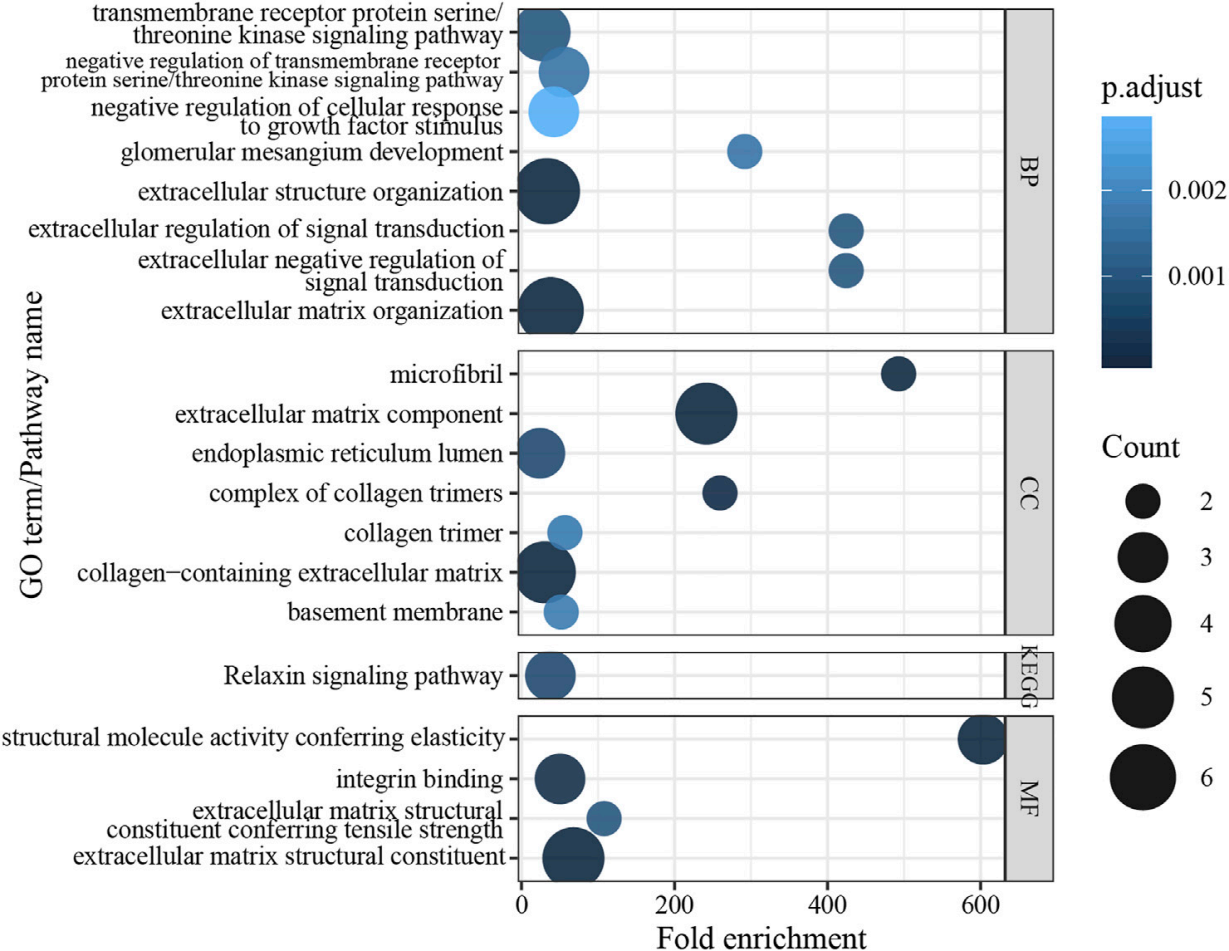
Results of enrichment analysis in GO term and KEGG pathway.

In order to understand the functional roles behind the mutation genes in TAAD biology, functional enrichment analysis was performed for gene function annotation *in silico*. We performed GO and KEGG enrichment analyses to uncover specific functional categories of the eight genes. Since *COL3A1* is related to aortic and arterial aneurysms, it was also included in the enrichment analysis. As a result, the eight genes clustered most significantly in 285 GO functional categories and four KEGG pathways (Figure 4, *p* values <0.05 after Benjamini adjustment). This analysis revealed that most of the identified genes encode proteins related to the extracellular matrix, kidney development, or relaxin signaling pathways. These results suggest that the variation in these genes might induce extracellular matrix degradation and force a decrease in fibrosis in TAAD biology.

## Discussion

In the current study, we examined three Chinese families with TAAD by screening for mutations in the 160 TAAD-causing genes list. We concluded that compound heterozygous mutations of *COL3A1*, *ACTA2*, *FBLN5*, *FBN1*, *SLC2A10*, *FBN2*, and *NOTCH1* are related to the familial TAAD. Furthermore, the bioinformatics analysis uncovered that these genes are involved in extracellular matrix organization. However, it was difficult to determine whether the mutation is a hereditary mutation in the pedigree since we just observed the disease’s natural course of partial TAAD individuals. Considering the inheritance pattern and damaging score, we focus on the *de novo COL3A1* mutations in TAAD-1 and *de novo ACTA2* mutations in TAAD-3. The data provide direct evidence that mutations of *COL3A1* and *ACTA2* are probably the major cause of familial TAADs in TAAD-1 and TAAD-3, respectively.

The human *COL3A1* gene comprises 51 exons and encodes the pro-alpha1 chains of type III collagen. Type III collagen constitutes about 5–20% of the entire collagen content in the human body that is found in extensible connective tissues such as the lung, skin, and the vascular system (Ruscitti et al., 2021). The first mutation of *COL3A1* was described in an inherited Ehlers-Danlos syndrome type IV patient in 1988 (Superti-Furga et al., 1988). Many mutations have also been reported to be associated with TAAD (Kontusaari et al., 1990a; Kontusaari et al., 1990b; Gu et al., 2018; Amitai Komem et al., 2019). Mutations in this gene cause a variety of vascular diseases, such as familial TAAD, Ehlers-Danlos syndrome, and cardiovascular phenotype. The mutation found in this study results in the replacement of base 2753 by G to A on exon 39 of chromosome 2, resulting in the conversion of amino acid 918 from glycine to glutamate. The Gene Ontology results show that *COL3A1* is involved in molecular function extracellular matrix structural constituent and extracellular matrix structural constituent, conferring tensile strength. The molecular function indicated that the mutation affects the aorta smooth muscle tissue morphogenesis.

The human *ACTA2* gene comprises nine exons and encodes one of six different actin proteins. Actins are highly conserved proteins that are involved in cell motility, structure, and integrity. It encodes the smooth muscle actin involved in vascular contractility and blood pressure homeostasis. Mutations in *ACTA2* cause a variety of vascular diseases, including thoracic aortic disease, coronary artery disease, stroke, and multisystemic smooth muscle dysfunction syndrome. Mutations in *ACTA2* were first reported in 2007 (Guo et al., 2007). Since then, many additional mutations have been reported in different populations (Yoo et al., 2010; Hoffjan et al., 2011; Malloy et al., 2012; Roque Rodriguez et al., 2020). We reviewed the literature and found that it has yielded *ACTA2* exon 6 mutations in the TAAD Caucasian populations (Meuwissen et al., 2013). This reminds us that more attention should be paid to exon 6 when screening for mutations causing *ACTA2* in TAAD families. Moreover, the corresponding protein sequence encoded by exon 6 is likely to be the key functional region of *ACTA*.

In summary, this study adds novel mutation loci to the existing spectrum of *COL3A1*, *ACTA2*, *FBLN5*, *FBN1*, *SLC2A10*, *FBN2*, and *NOTCH1* mutations with TAAD. Considering familial TAAD, the family history and the disease course in the family members may impact the future planning of TAAD individuals. Thus, screening of family members who have the same mutations but have not developed the disease is essential. From a genetic counseling point of view, we would inform the family with similar mutations of the TAAD possibility in the subsequent child. Judging from the current sequencing results, we speculate that the occurrence of the familial TAAD clinical phenotype requires some incentives, such as the weak with age or unmanageable blood pressure. From the genetic counseling perspective, family members who did not carry the same mutation and developed the disease will be informed of the risk of TAAD in offspring with similar mutations. This work represents significant steps for the selection of genes in hereditary TAAD. Subsequent studies are needed to provide new insights into this inherited disease.

## Data Availability

The data presented in the study are deposited in the National Genomics Data Center, accession number PRJCA008829.
